# Submission of structural biology data for review purposes

**DOI:** 10.1107/S2052252521012999

**Published:** 2021-12-16

**Authors:** Edward N. Baker, Charles S. Bond, Elspeth F. Garman, Janet Newman, Randy J. Read, Mark J. van Raaij

**Affiliations:** a School of Biological Sciences, University of Auckland, Auckland, New Zealand; b University of Western Australia, 35 Stirling Highway, Crawley, WA 6009, Australia; c University of Oxford, South Parks Road, Oxford OX1 3QU, United Kingdom; d Collaborative Crystallisation Centre (C3), CSIRO, 343 Royal Parade, Parkville, VIC 3052, Australia; e School of Biotechnology and Biomolecular Sciences, UNSW, Sydney, NSW 2052, Australia; f Cambridge Institute for Medical Research, University of Cambridge, The Keith Peters Building, Hills Road, Cambridge CB2 0XY, United Kingdom; g Departamento de Estructura de Macromoleculas, Centro Nacional de Biotecnologia, Consejo Superior de Investigaciones Cientificas, E-28049, Madrid, Spain

**Keywords:** structural biology, data, peer review

## Abstract

The editors discuss the submission of structural biology data.

The IUCr stable of journals are proponents of the FAIR and FACT principles of publishing: data should be Findable, Accessible, Interoperable and Reusable (Wilkinson *et al.*, 2016 ▸) and Fair, Accurate, Confidential and Transparent (van der Aalst *et al.*, 2017 ▸; Helliwell, 2019 ▸). This has been manifested by a number of leading contributions to the field of structural biology including publication standards for a variety of data types (Kroon-Batenburg & Helliwell, 2014 ▸; Adams *et al.*, 2019 ▸; Guss & McMahon, 2014 ▸) as well as mandatory deposition of coordinates, and subsequently structure factors, for publication of macromolecular crystal structures. This approach has largely satisfied the FAIR and FACT principles. However, a further step is required to ensure that the data deposited in a third-party database fully support the conclusions drawn from those data. This step requires the mandatory submission of data (*e.g.* coordinates and reduced diffraction data, discussed further below) to accompany the manuscript describing the work.

This action does not imply that the various databases [PDB (Burley *et al.*, 2019 ▸), EMDB (Lawson *et al.*, 2016 ▸), SASBDB (Kachala *et al.*, 2016 ▸), BMRB (Ulrich *et al.*, 2008 ▸) *etc.*] are not doing a good job of validating data submitted to them. Indeed, the validation reports produced by databases are an important pillar in the FAIR approach to structural biology. Nevertheless, it is not the role of the databases to test the extent to which the interpretations and conclusions of a manuscript incorporating structural biology research (possibly by multiple methods) are supported by the deposited data. This role belongs in the manuscript review cycle.

Authors may ask why submission of data needs to be made mandatory. After all, the Notes for Authors for *Acta Cryst. D*, *Acta Cryst. F* and *IUCrJ* all indicate that editors may request such data from the author. However, the current system causes delays in the review system when editors have to request data. The added inconvenience increases the possibility of poorly supported interpretations avoiding scrutiny and being published. In order to clarify the situation, and maintain the IUCr’s position leading the field in data quality standards in structural biology, submission of deposited data for review is best practice.

Historically there has been reticence to provide data for review as this might provide a competitor with an advantage. This concern has reduced substantially over recent years as increasing numbers of macromolecular structures are released prior to publication, and complementary methods such as accurate and precise computational predictions are more widely used. Indeed, the increasing prevalence of preprint versions of papers has further expedited the release of non-peer-reviewed data. Data submitted to IUCr Journals will be mandated *for review purposes only*, and thus the ethical position of a reviewer receiving them is clearly defined.

The concept of data deposition can be extremely broad, so it is important that we clarify what is mandated and what is recommended. The non-negotiable starting point is that for any manuscript containing conventional structures determined by the most common techniques (crystallography, NMR, cryoEM, SAXS) the data that are deposited with the relevant database to obtain the accession code and validation reports must be uploaded prior to editorial review. This set of standard files (which may include coordinate files, reduced diffraction data, restraint lists, electron-density maps, buffer-subtracted scattering curves, along with any validation reports) should already be immediately at hand to the submitting author. Additionally, depending on the manuscript in question there are cases where additional data should be provided. For example, many crystal structures may have indications of space-group ambiguity, twinning, anisotropy, alternate unit cells or other pathologies for which the review process would benefit from the submission of scaled but unmerged intensity data. We do not mandate submission for review of primary data as this is not practicable, however we continue to recommend that such data be made publicly available (Guss & McMahon, 2014 ▸).

To accompany the requirement for data, which will apply from the start of 2022, the journals *Acta Cryst. D*, *Acta Cryst. F* and *IUCrJ* are unifying their requirements for information to be included in the standard tables for various experiment types (colloquially Table 1 for crystal structures). These new requirements will be described in detail in the revised Notes for Authors for the journals.
